# Update from the National Hypertension Taskforce of Australia: prevalence, treatment and control rates of hypertension between 2022 and 2024 — implications and future directions

**DOI:** 10.1097/HJH.0000000000004358

**Published:** 2026-05-27

**Authors:** Amelie H. Ohlrogge, Xiaoyue Xu, Andrew Goodman, Garry Jennings, Lisa Murphy, Mark R. Nelson, James E. Sharman, Rob Tassie, Markus P. Schlaich, Aletta E. Schutte

**Affiliations:** a The George Institute for Global Health,; b School of Population Health, University of New South Wales, Sydney, Australia; c Department of Cardiology, University Heart and Vascular Center Hamburg; d German Center for Cardiovascular Research (DZHK), partner site North, Hamburg, Germany; e Australian eHealth Research Centre, Commonwealth Scientific and Industrial Research Organisation (CSIRO); f School of Public Health, The University of Queensland, Turrbal, Jagera Country, Brisbane; g Heart Foundation, Baker Heart and Diabetes Institute, Monash University, Melbourne; h The University of Sydney, Sydney; i Stroke Foundation, Melbourne; j Menzies Institute for Medical Research, University of Tasmania, Hobart; k Australian Cardiovascular Alliance, Sydney; l Dobney Hypertension Centre, Royal Perth Hospital and Royal Perth Hospital Research Foundation, University of Western Australia; m Departments of Cardiology and Nephrology, Royal Perth Hospital, Perth, Australia

**Keywords:** Australia, control, hypertension, National Hypertension Taskforce, population, prevalence

## Abstract

High blood pressure is the dominant risk factor for cardiovascular diseases and death in Australia. The blood pressure control rate in Australia was reported at 32% based on 2017–2018 data, which is substantially lower than other high-income countries. New results from the National Health Survey show an improvement of the crude control rate to 39.6% in the years 2022–2023, though changes in methodology need to be considered in the interpretation. Hypertension prevalence has increased from 33.7 to 39.4%. The prevalence and control rates vary strongly across diverse population groups. The age-standardized prevalence among First Nations peoples is higher than in the general population (37.8 versus 35%), and age-standardized blood pressure control rates are lower (22.2 versus 31.1%). Younger individuals and those in socioeconomically disadvantaged areas have substantially lower control rates, highlighting the need for greater equity to achieve the National Hypertension Taskforce's goal of 70% blood pressure control by 2030.

## INTRODUCTION

High blood pressure (BP) is the leading risk factor for cardiovascular diseases (CVD) and premature death in Australia [1,2]. The BP control rate in Australia was reported as 32% in 2017–2018 based on the conservative target of less than 140/90 mmHg [3]. This BP control rate is substantially lower than in other high-income countries, such as Canada and South Korea [4]. Antihypertensive treatment is highly effective for the prevention of CVD and highly cost-effective in the Australian healthcare system [5]. The National Hypertension Taskforce of Australia was founded in 2022 with the goal to improve BP control rate to 70% by 2030 [3].

On 15 May 2026, the most recent results from the National Health Survey (NHS) were published by the Australian Institute of Health and Welfare (AIHW). This nationwide cohort provides the most up-to-date key indicators for BP control in Australia. Here we interpret this data in context of the goals set out in the Roadmap of the National Hypertension Taskforce [3].

## METHODS

The NHS is a representative nationwide Australian cohort. Households were randomly selected, and 13 095 of these households (56.7%) with 17 073 individuals participated between 2022 and 2023. In the National Health Measures Survey, health-related measures were collected between 2022 and 2024, including measured BP. The data collection for antihypertensive medication changed from previous iterations and is now linked to dispensing data of the government's Pharmaceutical Benefits Scheme (PBS), including the dispensing of antihypertensive medication 6 months before and after study enrolment instead of self-reported medication use in the last 2 weeks. Hypertension is defined as a measured SBP or DBP of at least 140 mmHg and/or at least 90 mmHg and/or the use of antihypertensive medication. Controlled BP is defined as individuals with measured BP less than 140 mmHg and less than  90 mmHg while using antihypertensive medication. First Nations peoples were examined in a separate cohort, the 2022–2023 National Aboriginal and Torres Strait Islander Health Survey (NATSIHS). In this cohort, 7839 households were randomly selected, and 4878 households with a total of 7768 individuals participated. Further methods are reported elsewhere [6–8].

## RESULTS

**FIGURE 1:**
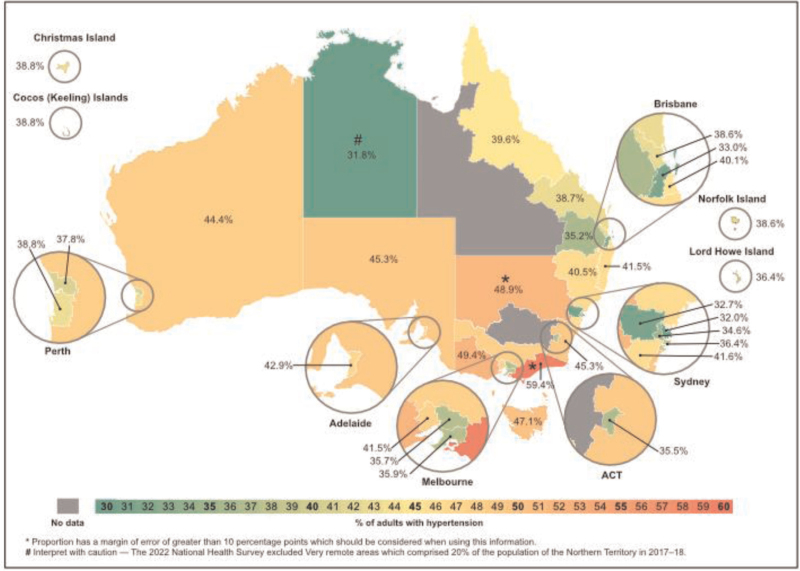
Crude prevalence of hypertension among adults in 2022–2023 by Primary Health Network (PHN) area. ^▪^Proportion has a margin of error of greater than 10 percentage points, which should be considered when using this information. ^#^Interpret with caution – The 2022 National Health Survey excluded very remote areas which comprised 20% of the population of the Northern Territory in 2017–2018. Data Source: ABS 2022 National Health Survey.

**FIGURE 2:**
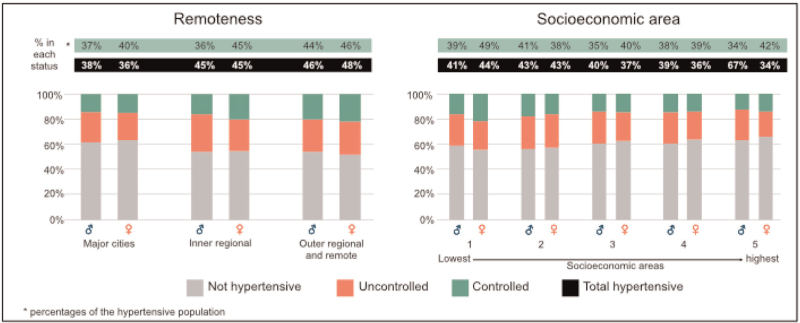
Crude prevalence of hypertension and BP control rates among adults in 2022–2023 by remoteness category and socioeconomic area. Rates for controlled and uncontrolled BP are given as percentages of the total population with hypertension. Data source: ABS 2022 National Health Survey.

Of the Australian population aged 18 years and over, the crude prevalence of hypertension was 39.4% with large regional differences (Fig. 1). This translates to approximately 7.2 million adults with hypertension. Males had higher rates of hypertension than females (40.1 versus 38.7%). Hypertension prevalence increased with remoteness (from 37.3% in major cities to 46.7% in outer regional and remote areas), particularly for females. However, this effect was partially attenuated by age-standardization (numbers not displayed, see AIHW publication) [9]. Hypertension prevalence was the highest in disadvantaged socioeconomic areas. Again, this discrepancy was larger for females and partially ameliorated by age-standardization. The BP control rates were lower in major cities (37–40%) than in outer regional and remote areas (44–46%) (Fig. 2).

**FIGURE 3:**
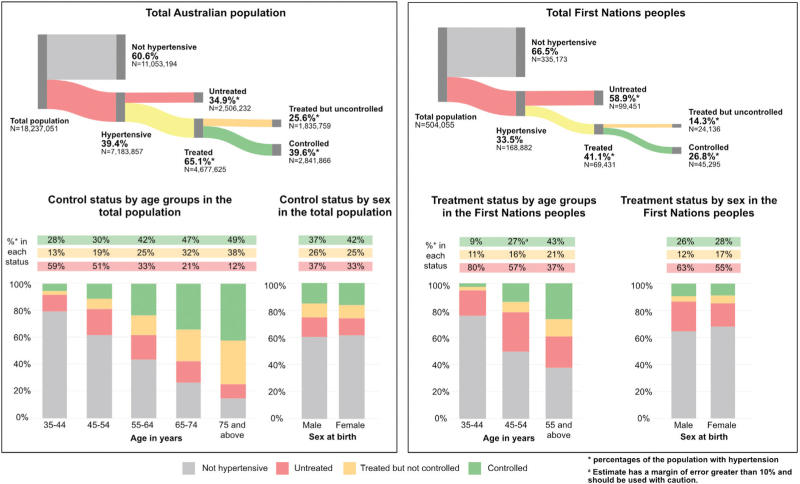
Crude blood pressure control and hypertension treatment percentages in the Total Australian and First Nations peoples in 2022–2023. The prevalence of hypertension is given as a percentage of the total population; the BP control and treatment are given as percentages of the population with hypertension. Note that the estimate for BP control in the age group 45–54 years in First nations peoples has a margin of error greater than 10% and should be used with caution. Fewer age groups were available for First Nations peoples in the dataset. The total numbers indicated in the flow chart are estimates for the total Australian population. Data Source: ABS 2022 National Health Survey, ABS 2022–2023 National Aboriginal and Torres Strait Islander Health Survey.

The prevalence of hypertension increased with age in both sexes (Fig. 3). Rates of antihypertensive treatment were lowest in the youngest age group (18–34 years, 22.8% treatment) and increased with age (87.7% in those 75 years and above). Females were treated more often than males (66.9 versus 63.3%).

BP control (<140/90 mmHg) was 39.6% (age-standardized 31.3%) overall, and higher among females than males (41.7 and 37.4%). Control rates were lowest in the youngest age group (18–34 years), particularly in males (13.3% in males and 23.7% in females). These sex differences in control rates were less pronounced with increasing age (Fig. 3).

Compared to the total population, hypertension prevalence was overall lower in First Nations peoples (Fig. 3). However, the age-standardized rates of hypertension are higher (37.8 versus 35%) than in the total population (Supplement Figure 1). Treatment and BP control rates in the First Nations peoples were considerably lower than in the total population (age-adjusted BP control rates 22.2% in First Nations peoples versus 31.1% in the total population), most pronounced in younger age groups and in males.

There is a positive trend in the control rates in the total population compared to earlier years from 32.1% (2017–2018) to 39.6% (2022–2023), though the hypertension prevalence increased (from 33.7 to 39.4%). However, an opposite trend can be observed among First Nations peoples. Here, the control rate fell from 26.5% (2018–2019) to 22.2% (2022–2023). The age-standardized rates of a measured high BP increased only in the First Nations peoples from 25.1 to 26.6% but not in the total population, most pronounced in males (26.5–29%) (Supplement Figure 1).

## DISCUSSION

This dataset provides a valuable update on the prevalence, treatment and control rates of hypertension in Australia from the largest representative national cohort. Compared to 2017–2018, the hypertension prevalence in the general population has increased from 33.7 to 39.4%, reflecting an estimated total of more than seven million Australians. The recent crude BP control rate was 39.6%, an almost 8% increase in BP control from 32.1% in 2017–2018, comparable to the average of high-income countries [4,10]. This is perhaps a promising signal that the national efforts to improve BP control are starting to make a difference, though there is still a significant gap before the Hypertension Taskforce's goal of achieving 70% BP control by 2030 can be met. At the current rate of improvement, BP control will barely exceed 50% by 2030. A key challenge in Australia and globally remains that BP control in global studies, including this one, is based on the conservative threshold of less than 140/90 mmHg. Instead, BP should be lowered to less than 130/80 mmHg for optimal cardiovascular protection. If this lower threshold were to be applied to the current analysis, the BP control rates will be much lower.

This data identifies key areas that require attention. These include lower BP control rates among: First Nations peoples, in which BP control rates have worsened since 2018–2019; those in socioeconomically disadvantaged areas; and younger individuals (aged 18–54 years), in particular younger men. It should be noted that in the last group, secondary causes of hypertension and uncertainties on the benefit of antihypertensive treatment due to lower short-term cardiovascular risk may play a role [11], though lifetime cardiovascular risk is increased also in these young individuals [12]. Intensified efforts are needed to specifically target these three underserved populations and improve healthcare equity.

Some limitations need to be acknowledged. Even though selection of households was random in the National Health Survey, only 56.7% participated. Though hypertension awareness is reported, the definition relies on self-reported hypertension. This was implausibly lower than the treatment rates and is therefore very unlikely to reflect true diagnosis rates. First Nations peoples living in remote and very remote areas have been excluded from this analysis, thereby the potentially most disadvantaged individuals are not reflected in this dataset [13]. Temporal comparisons are difficult since the data collection of antihypertensive medication use changed from self-reported use to PBS data. This may have partly affected the increase in hypertension prevalence and affected control rates compared to earlier iterations, as likely more people with normal measured BP will be classified as having treated and controlled hypertension. Despite these limitations, this dataset remains the most reliable source for the assessment of hypertension in the Australian general population, which is urgently needed to track trends.

**FIGURE 4:**
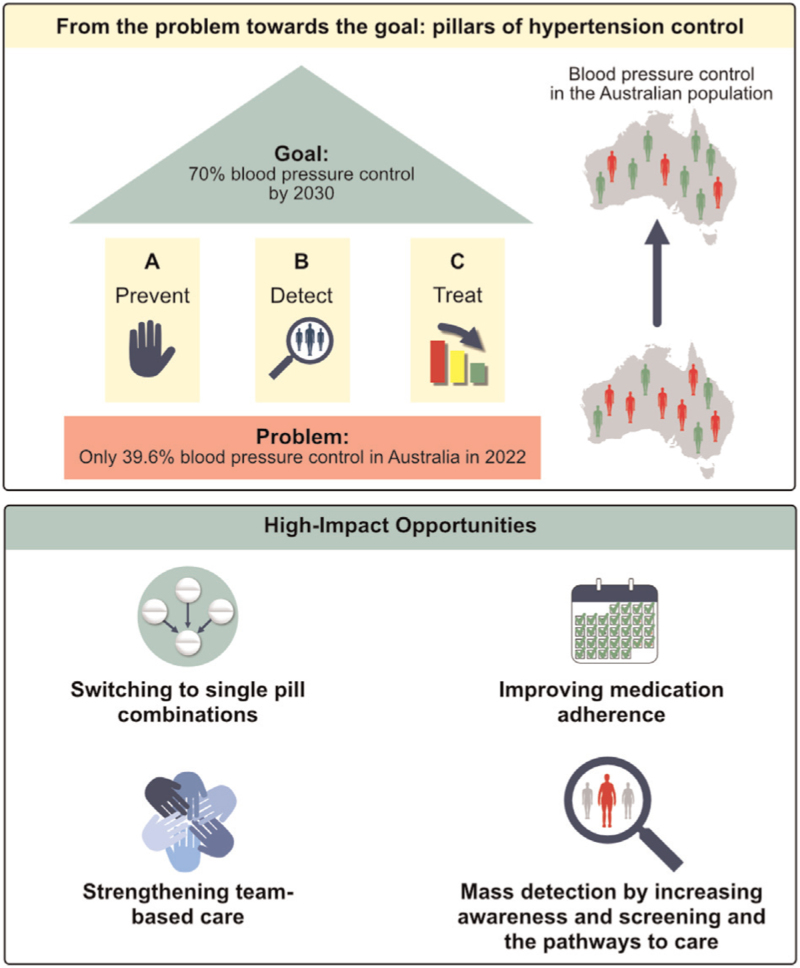
Summary of the Hypertension Taskforce goal, pillars and high-impact opportunities.

Even though there has been some improvement compared to previous reports, control rates still need to almost double to achieve the 70% goal of the National Hypertension Taskforce. The actions required to achieve this are built on three pillars: Prevent, Detect, Treat effectively [3]. Key areas for prevention on a population level are the reduction of sodium intake, for example, through legislation reform on processed food or the substitution or regular salt with potassium-enriched alternatives [14]. Four specific areas of high-impact opportunities have since been highlighted for detection and effective treatment: switching to single pill combination therapy; improving medication adherence; strengthening team-based care; and mass detection by increasing awareness, screening and the pathways to care (Fig. 4). Efforts in these areas must intensify further to reduce hypertension-related morbidity and mortality. Improving BP control in Australia could not only save AU$91.6 billion but also save many thousands of lives [3].

## ACKNOWLEDGEMENTS

We thank the members of the AIHW, in particular Dian Xu, Doreen Busingye, Kate Hafekost and Heidi Dietz, for their review of this manuscript and collaboration on this project.

The data presented in this work is separately published by the Australian Institute of Health and Welfare online on their website.

### Conflicts of interest

A.E.S. has received speaker honoraria from Servier, Sanofi, AstraZeneca, Medtronic, Omron, Novo Nordisk, and Sky Labs and serves on scientific advisory boards for Medtronic, AstraZeneca, Servier, and Sky Labs. M.S. has received speaker fees/travel support/conference support from Medtronic, ReCOR, Abbott, Astra Zeneca, Servier, NovoNordisk, Amgen, Boehringer Ingelheim, and serves on advisory boards for Abbott, Medtronic, Astra Zeneca, Idorsia, Kardigan, and Boehringer Ingelheim.

## Supplementary Material

Supplemental Digital Content
