# Supplementary material for: Update from the National Hypertension Taskforce of Australia: prevalence, treatment and control rates of hypertension between 2022 and 2024 — implications and future directions
Source: J Hypertens. 2026 May 27;44(8):1457–61. doi: 10.1097/HJH.0000000000004358 (PMC13336605; doi:10.1097/HJH.0000000000004358)
Supplement: Supplemental Digital Content [file jhype-44-1457-s001.docx]

**Supplement**

*Update from the National Hypertension Taskforce of Australia:*

*Prevalence, treatment and control rates of hypertension between 2022-2024 – implications and future directions*


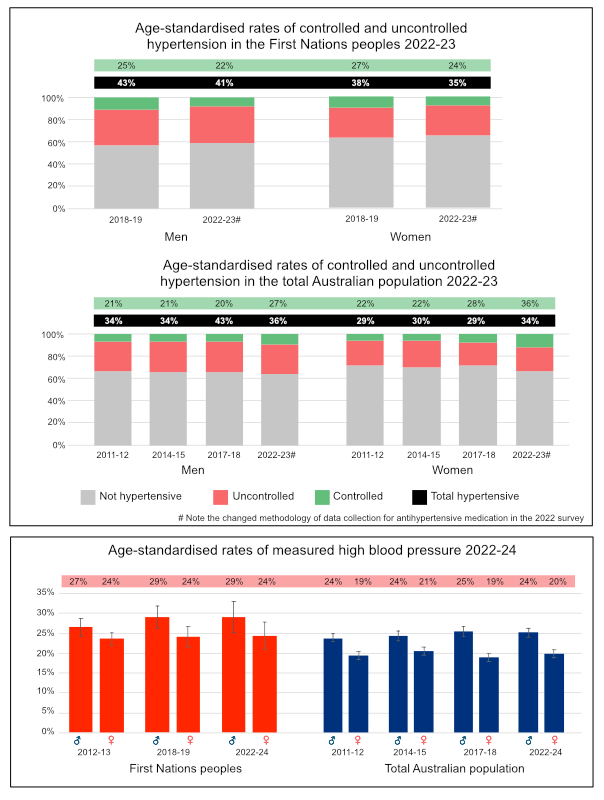


**Supplement figure 1** - Age-standardised rates of controlled and uncontrolled hypertension in the First Nations and total Australian population (upper panel) and age-standardised rates of measured high blood pressure in the First Nations peoples and total Australian population (lower panel). #Note the changed methodology of data collection for antihypertensive medication in the 2022 survey. Data Source: ABS 2022 National Health Survey, ABS 2022-23 National Aboriginal and Torres Strait Islander Health Survey, ABS 2022-24 National Health Measures Survey
